# Enhanced Performance and Durability of Pore-Filling Membranes for Anion Exchange Membrane Water Electrolysis

**DOI:** 10.3390/membranes14120269

**Published:** 2024-12-12

**Authors:** Minyoung Lee, Jin-Soo Park

**Affiliations:** 1 Department of Green Chemical Engineering, College of Engineering, Sangmyung University, Cheonan 31066, Republic of Korea; 2 Future Environment and Energy Research Institute, Sangmyung University, Cheonan 31066, Republic of Korea

**Keywords:** pore-filling membrane, anion exchange membrane, anion-conducting electrolyte, water electrolysis, hydrogen

## Abstract

Four distinct pore-filling anion exchange membranes (PFAEMs) were prepared, and their mechanical properties, ion conductivity, and performance in anion exchange membrane water electrolysis (AEMWE) were evaluated. The fabricated PFAEMs demonstrated exceptional tensile strength, which was approximately 14 times higher than that of the commercial membrane, despite being nearly half as thin. Ion conductivity measurements revealed that acrylamide-based membranes outperformed benzyl-based ones, exhibiting 25% and 41% higher conductivity when using crosslinkers with two and three crosslinking sites, respectively. The AEMWE performance directly correlated with the hydrophilicity and ion exchange capacity (IEC) of the membranes. Specifically, AE_3C achieved the highest performance, supported by its superior IEC and ionic conductivity. Durability tests showed that AE_3C outlasted the commercial membrane, with a delayed voltage increase corresponding to its higher IEC, confirming the importance of increased ion-exchange functional groups in ensuring longevity. These results highlight the critical role of hydrophilic monomers and crosslinker structure in optimizing PFAEMs for enhanced performance and durability in AEMWE applications.

## 1. Introduction

Hydrogen stands out as an environmentally friendly energy carrier that aids in achieving carbon neutrality and driving the transition to a low-carbon economy [1,2,3,4,5,6,7,8]. As a way to reduce the reliance on fossil fuels and decrease pollution, hydrogen production via renewable-powered water electrolysis is gaining attention. Key technologies in this field include alkaline water electrolysis (AWE), proton exchange membrane water electrolysis (PEMWE), and anion exchange membrane water electrolysis (AEMWE), which are widely utilized across research and industrial sectors [9,10,11]. AEMWE uniquely combines the benefits of AWE and PEMWE, employing an anion exchange membrane to achieve high-purity hydrogen similar to PEMWE while using cost-effective catalysts like those in AWE. To further improve hydrogen production systems, advancements in material design, component refinement, and system integration are essential for enhancing efficiency and durability [12].

Anion exchange membranes (AEMs) play a vital role in technologies that require the effective conduction of hydroxide ions (OH^−^). These membranes are composed of anion-conducting polymers (ACPs), featuring positively charged cationic head groups to support OH^−^ ion transport. To perform optimally, AEMs must demonstrate both high ion conductivity and strong mechanical resilience. Several methods have been devised to improve these traits, including crosslinking, microphase separation, and composite structuring. Crosslinking strengthens connections between the anion-conducting polymer chains, which enhances both the membrane’s mechanical stability and resistance to alkaline conditions, helping it retain its structure and ionic conductivity during operation [13,14,15,16,17,18,19,20]. Microphase separation creates distinct regions within the membrane that differentiate water-attracting (hydrophilic) and water-repelling (hydrophobic) areas, organizing polymer side-chains and backbones in a dual-phase arrangement that improves ionic movement. This design supports well-distributed ionic domains, boosting both conductivity and mechanical strength [21,22,23]. Lastly, composite membranes incorporate inorganic nanoparticles into the ACPs or reinforce the membranes with porous or woven materials. These enhancements elevate ion conductivity, control swelling, and improve the membrane’s durability, making it more adaptable for various applications [24,25,26,27,28,29,30].

New methods focus on enhancing both the mechanical and chemical durability of materials while enabling higher operating temperatures. Pore-filling membranes, formed by infusing porous substrates with polymer or monomer electrolytes, show promise in achieving these goals. These substrates are generally hydrophobic polyolefins characterized by high porosity and small pore sizes. Within these membranes, ion transport mainly occurs through the solidified, pore-filled ACP, with its chemical composition playing a crucial role in determining ionic conductivity. Thus, carefully selecting compatible electrolyte monomers and crosslinking agents is key to optimizing performance [31,32,33].

The properties of ACP within the porous substrate vary significantly depending on the combination of electrolyte monomer and crosslinker, ultimately influencing the overall membrane characteristics [22,28,29,31,32,33]. In this study, two types of electrolyte monomers—one based on acrylamide and the other on benzyl groups—and two types of crosslinkers, containing either two or three vinyl groups, were selected to create four distinct types of pore-filling membranes. The acrylamide-based electrolyte monomer is more hydrophilic than the benzyl-based monomer because the aromatic ring in the benzyl group enhances hydrophobicity by increasing the affinity for non-polar environments. Additionally, a crosslinker with a greater number of vinyl groups provides more functional groups per unit weight of ACP, thereby increasing the ion exchange capacity (IEC) of the ACP. Finally, the effects of four different pore-filling membranes, produced using the two electrolyte monomers and two crosslinking agents mentioned above, were examined with respect to their performance and durability in water electrolysis.

## 2. Materials and Methods

### 2.1. Porous Substrate and Hydrophilization

A 25 µm thick porous polyethylene (PE) support with 40% porosity and 70 nm pore size was used as the base material. Sodium dodecylbenzenesulfonate, an anionic surfactant from Sigma-Aldrich (Darmstadt, Germany), was applied during the pretreatment process since the porous PE is inherently hydrophobic. A hydrophilization step was necessary to modify the PE surface. A 0.5 wt.% solution of the surfactant in distilled water was prepared for this purpose. The substrate was initially washed with ethanol, dried completely at room temperature, and then soaked in the surfactant solution to facilitate the hydrophilization. Afterward, it was dried again at room temperature to finalize the treatment.

### 2.2. Preparation of Pore-Filling Membranes

The electrolyte monomers used to prepare the pore filling anion-exchange membrane (PFAEM) were (3-acrylamidopropyl)trimethylammonium chloride (75 wt.% in H_2_O) and (vinylbenzyl)trimethylammonium chloride (99%), both from Sigma-Aldrich (Darmstadt, Germany). The crosslinkers were 1,4-bis(acryloyl)piperazine (≥99.0%) and trimethylolpropane trimethacrylate, also sourced from Sigma-Aldrich (Darmstadt, Germany). As a photoinitiator, 2-hydroxy-2-methylpropiophenone (97%, Sigma-Aldrich, Darmstadt, Germany) was used. Table 1 provides the chemical structures, names, and molecular weight of the electrolyte monomers and crosslinkers. The electrolyte monomer (3-acrylamidopropyl)trimethylammonium chloride, containing an acrylamide group, was designated AE, while (vinylbenzyl)trimethylammonium chloride, containing a benzene group, was labeled BE. Crosslinkers were named according to the number of crosslinking sites: 1,4-bis(acryloyl)piperazine (with two sites) as 2C, and trimethylolpropane trimethacrylate (with three sites) as 3C.

**Figure 1:**
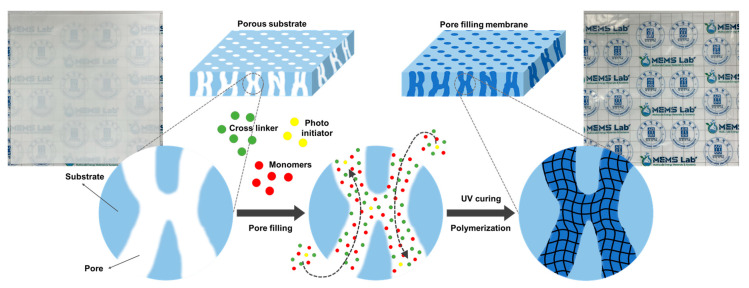
Schematic diagram for the preparation of pore-filling membranes.

Crosslinker 2C, which contains two crosslinking sites, was used as a solvent in ethanol to achieve a solid content of 20 wt.%, while BE, an electrolyte monomer, was used with ethanol to reach a solid content of 50 wt.%. The solutions were mixed in molar ratios of electrolyte to crosslinker of 24:1. The photo-initiator was prepared as a 10 wt.% solution in ethanol. Four to five drops (0.08 g) of the photo-initiator solution were added to the thoroughly mixed electrolyte and crosslinker solution, and the mixture was stirred using a magnetic stirrer. During this process, the reagent bottle was wrapped in foil to minimize light exposure. The hydrophilic porous substrate was fully immersed in the mixed solution for 20 min. Once the monomer filled the substrate’s pores, the substrate was sandwiched between polyethylene terephthalate (PET) films, and the solution was removed by applying constant pressure. Photo-polymerization was carried out for 15 min using a UV curing device (ramp power = 1.5 kW). After the polymerization, the PET films were removed, and the PFAEM was washed with distilled water and left to dry at room temperature. The conceptual process for preparing the PFAEM, along with the appearance of the porous substrate and the PFAEM, is illustrated in Figure 1. Four different PFAEMs, AE_2C, AE_3C, BE_2C, and BE_3C, were prepared for the characterization and the evaluation of AEMWE performance and durability.

### 2.3. Characterization of Membranes

Fourier transform infrared (FT-IR) is an analytical instrument that modifies the wavelength of light within the infrared spectrum to measure the absorption intensity of energy corresponding to the unique vibrational and rotational movements of a specific substance when exposed to light. By analyzing the absorption levels at various wavenumbers, FT-IR can identify a substance’s chemical structure and provide details on functional groups or molecular motion present. In this study, an FT-IR spectrometer (Spectrum 100, PerkinElmer, Waltham, CA, USA) was utilized to confirm the combination of anionic conductive monomers with crosslinking monomers, as well as to characterize the overall chemical structure of the AEPFM. Measurements were conducted within a wavenumber range of 500–4000 cm^−1^.

To evaluate the mechanical properties of the AEPFM, membranes cut to 3 cm × 9 cm were prepared. The dried membranes’ properties were assessed using a universal testing machine (ST-1000, Salt, Incheon, Republic of Korea) following the ASTM D882 method.

For ion transport capacity measurement, 2 cm × 2 cm membrane samples were prepared and immersed in a 0.001 M NaCl solution for over 24 h, allowing the functional groups to be fully replaced by Cl^−^ ions and achieving equilibrium with the NaCl solution. Membranes were then positioned between two-compartment cells, with Ag/AgCl wire electrodes fixed near both ends. A 0.001 M NaCl solution was added on one side and 0.005 M NaCl on the other. After removing surface bubbles, the Ag/AgCl electrode was connected to a potentiostat/galvanostat (SP-150, BioLogics, Seyssinet-Pariset, France) to measure voltage. The ion transport number (*t_–_*) was determined by substituting the voltage into Equation (1) below:

(1) $$E_{m}\;(V)=\frac{RT}{F}\left({2t}_{-}-1\right)ln\frac{C_{1}}{C_{2}}$$

where *E_m_* (V) represents the voltage caused by the concentration difference, *R* is the gas constant (8.3145 J/mol·K), *T* (K) is the absolute temperature, *F* is the Faraday constant (96,485 C/mol), *t_–_* is the anion transport number, *C*_1_ is the 0.001 M NaCl, and *C*_2_ is the 0.005 M NaCl.

To determine the contact angle of AEPFMs, a membrane was cut to dimensions of 1 cm × 2 cm and immersed in a 1 M KOH solution for over 24 h to fully exchange the functional groups with OH^−^ ions. After soaking, residual KOH was carefully wiped from the membrane surface, which was then thoroughly air-dried at room temperature. A 3 μL droplet of distilled water was placed on the dried membrane as a sessile drop, and the contact angle was measured using a contact-angle-measuring device (TL 101, Theta Lite Optical, Biolin Scientific, Stockholm, Sweden) after a 10 s interval.

To measure the ion exchange capacity of the AEPFM, 2 cm × 2 cm membranes were prepared and placed in a 1 M KOH solution for 24 h, replacing functional groups with OH^−^ ions. The membranes were then rinsed with distilled water to remove residual KOH and immersed in a 0.01 N HCl solution for 24 h to substitute OH^−^ with Cl^−^. The membrane was then dried at 60 °C for 24 h, and its weight was measured. The 0.01 N HCl solution was titrated with 0.01 M NaOH using an automatic titrator (848 Titrino Plus, Metrohm, Herisau, Switzerland). The ion exchange capacity of AEPFM (IEC) was calculated using Equation (2) below:

(2) $$\mathrm{IEC}\;(\mathrm{meq}/g)=\frac{C_{NaOH}(V_{blank}-V_{memb})}{W_{dry}}$$

where *C_NaOH_* (M) is the NaOH molar concentration, *V_blank_* (mL) is the NaOH volume consumed for the titration of the blank, and *V_memb_* (mL) is the NaOH volume consumed for the titration of the membrane.

To measure the ionic conductivity of AEPFMs, a 2 cm × 2 cm membrane sample was prepared and immersed in a 1 M KOH solution for over 24 h to fully exchange functional groups with OH^−^ and achieve equilibrium with the KOH solution. The membrane thickness impedance was measured using a potentiostat/galvanostat (SP-150, Bio-Logic Science Instruments, Seyssinet-Pariset, France) in a clip cell setup at room temperature in the 1 M KOH solution. Impedance measurements were taken at frequencies ranging from 1 MHz to 1 Hz, with an applied strength of 20 mV. The membrane’s area resistance (*R_m_*) and ionic conductivity (*σ*) were calculated using Equation (3) below:

(3) $$\sigma \;(S/\mathrm{cm})=\frac{L}{R_{m}}=\frac{L}{{(R}_{s}-R_{b})A}$$

where *R_m_* (Ω·cm^2^) is the areal resistance of the samples, *R_s_* (Ω) is the impedance of the samples and the background, *R_b_* (Ω) is the impedance of the background (1 M KOH), *A* is the effective area of the samples (cm^2^), *σ* (S/cm) is the ionic conductivity, and *L* (cm) is the membrane thickness.

To assess the alkaline stability of AEPFM, membranes were prepared by cutting them into 2 cm × 2 cm pieces. These membranes were then placed in a 1 M KOH solution at room temperature for 24 h to ensure that all functional groups were fully substituted with OH^−^ ions, allowing equilibrium with the 1 M KOH solution. Following this, the membranes were removed from the solution, excess surface solution was wiped off, and they were immersed in a 4 M KOH solution at 60 °C for a period ranging from 0 to 400 h. The 4 M KOH solution was refreshed every 7 days. At each designated time point, the membranes were removed, and through-plane impedance was measured in a 1 M KOH solution at 60 °C to calculate the ionic conductivity using Equation (3).

Platinum on carbon (Pt/C) (TEC10F50E, Tanaka Kikinzoku Kogyo K.K., Tokyo, Japan) was used as a catalyst for the hydrogen evolution electrode, while Nafion D2021 (EW 1100, Chemours, Wilmington, DE, USA) served as the ionomer binder. Iridium oxide (IrO_2_) (43396, Alfa Aesar, Haverhill, MA, USA) was used as the catalyst for the oxygen evolution electrode, with Nafion D521 (EW 1100, Chemours, Wilmington, DE, USA) as the ionomer binder. The solid content of ionomer binders in the oxygen and hydrogen evolution electrodes was 20 wt.% and 30 wt.%, respectively. Deionized water, 1-propanol, and 2-propanol were added as solvents for both electrodes. Stirring was conducted using a magnetic stirrer for over 12 h. The prepared catalyst ink was coated onto a gas diffusion layer (carbon paper) (JNT20-A3, JNTG, Hwaseong, Korea) to create a hydrogen evolution electrode with an effective area of 9 cm^2^. It was also coated on a porous transport layer, titanium paper (2GDL09N-025, BEKAERT, Zwevegem, Belgium), to create an oxygen evolution electrode. The electrodes were fabricated by drying the solvent on a hot plate at 70 °C, using an automatic spray machine (Accumist™ Ultrasonic Spray Shaping, Sonotech, Halle, Germany). Catalyst loadings were 0.4 mg-Pt/cm^2^ for the hydrogen evolution electrode and 1.0 mg-Ir/cm^2^ for the oxygen evolution electrode, respectively. A 1 M KOH feed solution was supplied to the anodic chamber at a flow rate of 30 mL/min. The temperature of both the feed solution and the electrolyzer single cell was maintained at 60 °C.

The performance of the AEMWE was analyzed using the current–voltage (I–V) curve. The unit cell provided I–V data through a potentiostat (SP-150, Bio-Logic Science Instruments, Seyssinet-Pariset, France), with additional measurements obtained via electrochemical impedance spectroscopy. The current response was measured as the cell voltage was increased from 1.35 V to 2.0 V. After completing the electrolysis performance tests, a durability assessment was conducted by applying a constant current density of 0.5 A/cm^2^ with daily start–stop cycles using a power supply (PWR801L, Kikusui Electronics Corp., Yokohama, Japan).

For reference, the commercial AEM used was a Sustainion X37-50 Grade T (Dioxide Materials, Boca Raton, FL, USA).

## 3. Results and Discussion

**Figure 2:**
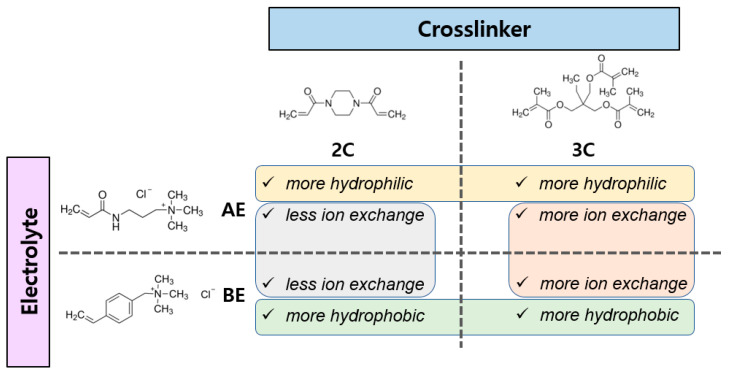
Schematic diagram for the preparation of pore-filling membranes.

In this study, four different PFAEMs, AE_2C, AE_3C, BE_2C, and BE_3C, were prepared by synthesizing ACPs crosslinking a relatively hydrophilic acrylamide-based electrolyte monomer and a relatively hydrophobic benzyl-based electrolyte monomer, each containing two and three vinyl groups, respectively. As a result, PFAEMs with varying levels of hydrophilicity and ion exchange capacity were prepared: those with high hydrophilicity and high (AE_2C) or low (AE_3C) ion exchange capacity, and those with high hydrophobicity and high (BE_2C) or low (BE_3C) ion exchange capacity. The membrane properties, AEMWE performance, and durability of these PFAEMs were evaluated. The characteristics of the four PFAEMs are summarized in Figure 2.

**Figure 3:**
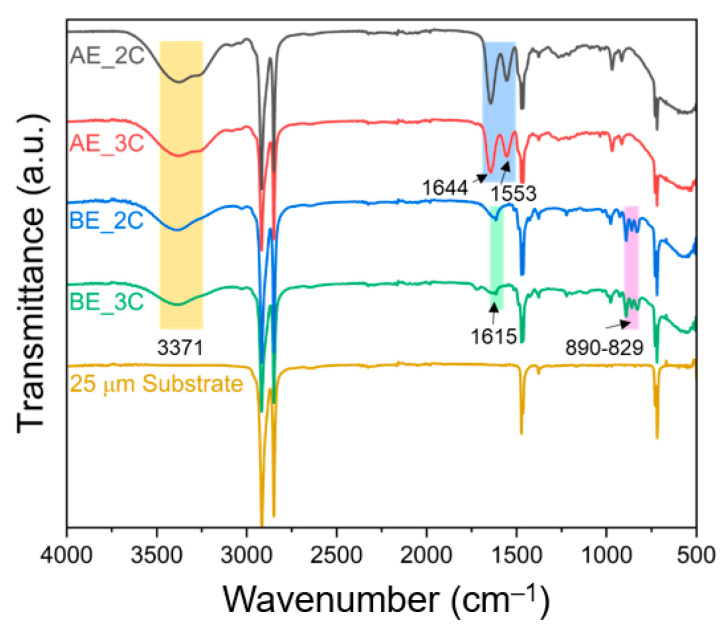
FT-IR spectra of PE substrate and four different AEPFMs.

Figure 3 presents the FT-IR spectra of both the PE substrate and the fabricated AEPFMs. PE is a polymer derived from the polymerization of ethylene (C_2_H_4_) monomers, forming chains of varying lengths (n values). In the PE substrate, four distinct peaks corresponding to the C-H asymmetric stretching, C-H symmetric stretching, CH_2_ scissoring, and CH_2_ rocking vibrations are detected at 2916, 2849, 1473, and 718 cm^−^¹, respectively [34]. These peaks are consistently observed across all AEPFM samples, confirming that the photo-polymerized material successfully filled the pores of the PE substrate. The spectrum of the PFAEMs differs significantly from that of the PE substrate. The OH stretching vibration is observed at a peak around 3371 cm^−1^, confirming the introduction of a quaternary ammonium functional group, a hydrophilic ion-exchange group, into all the PFAEMs. In the PFAEMs containing the AE electrolyte monomer, the amide I and amide II bands are observed at peaks of 1553 cm^−1^ and 1644 cm^−1^. In the PFAEMs containing the BE electrolyte monomer, the C-H bending vibration of the phenyl ring is seen at 890–829 cm^−1^ [35], while the C=C band of the benzyl group appears at 1615 cm^−1^ [36]. These observations confirm that the ACPs, formed by polymerizing the electrolyte monomers with the crosslinkers, are effectively bonded to the porous substrate.

**Figure 4:**
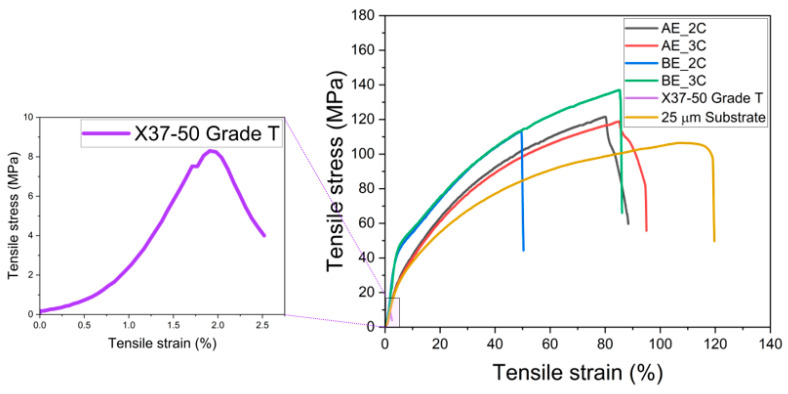
Stress–strain curves of PE substrate, PFAEMs, and commercial AEM.

Figure 4 presents the tensile strength of the fabricated PFAEMs, the commercial AEM, and the porous substrate. Table 2 provides comprehensive data on tensile strength and elongation at break. The fabricated PFAEMs exhibit tensile strengths between 113 and 137 MPa, which are significantly higher than the 8.3 MPa measured for the commercial membrane. This indicates that the tensile strength of the fabricated membranes is approximately 14 times greater than that of the commercial membrane. Notably, the PFAEMs, with a thickness of 28–30 µm, are nearly half as thin as the 50 µm X37-50 Grade T membrane; yet, they achieve superior mechanical strength due to the robust mechanical properties of the porous substrate. Additionally, the fabricated membranes display a lower elongation at break compared to the porous substrate, a result attributed to the effects of crosslinking [37]. It is confirmed that variations in the chemical structure of the ACP filled within the support do not influence the mechanical properties.

The ion transport number is a key parameter reflecting the ion selectivity of an ion exchange membrane, indicating how effectively the membrane rejects co-ions while selectively transmitting counter-ions. The results, presented in Table 3, reveal that the measured ion transport number is higher than that of the commercial AEM, X37-50 Grade T. All measured values are close to 1, confirming that the ionomer fully occupied the support pores, leaving no voids. This is because, if the ACP within the porous substrate is not completely filled, ions in the solution diffuse as paired cations and anions. Such paired diffusion limits the movement of counter-ions, thereby lowering the transport number. Thus, these results verify the successful fabrication of an AEM with high anion selectivity.

**Figure 5:**
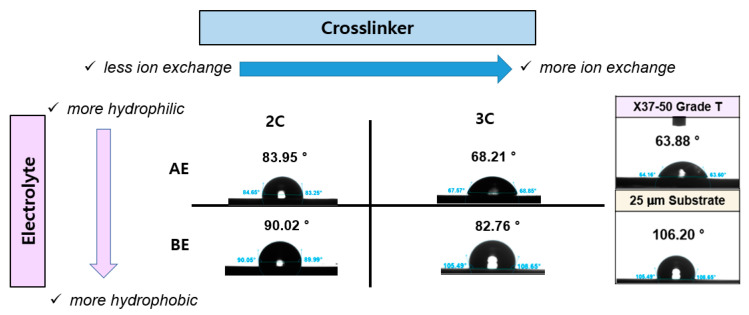
Contact angle of PE substrate, the PFAEMs, and the commercial AEM.

Figure 5 illustrates the contact angles of the PE substrate, the commercial AEM, and the PFAEMs. The commercial AEM, which is a homogeneous free-standing membrane, exhibits the highest surface hydrophilicity, with the smallest contact angle of 63.88°. In contrast, the four PFAEMs show higher contact angles compared to the commercial membrane but smaller angles than the hydrophobic PE support (106.20°) before hydrophilization, confirming that they are effectively filled by hydrophilic ACPs. Among the PFAEMs, the one using the 3C crosslinker with three crosslinking sites demonstrates a lower contact angle than the membrane using the 2C crosslinker with two crosslinking sites. Similarly, the membrane incorporating the hydrophilic electrolyte monomer AE exhibits a lower contact angle compared to the membrane using BE. This behavior can be attributed to the properties of the electrolyte monomer. The hydrogen bond acceptor number (HBA) represents the number of atoms in a molecule capable of forming hydrogen bonds. A higher HBA enables the formation of more hydrogen bonds, thereby enhancing the interaction with water and increasing hydrophilicity. Consequently, the HBA of the electrolyte monomer AE is higher than that of BE, and the HBA of the crosslinker 3C is higher than that of 2C, making AE more hydrophilic than BE and 3C more hydrophilic than 2C.

**Figure 6:**
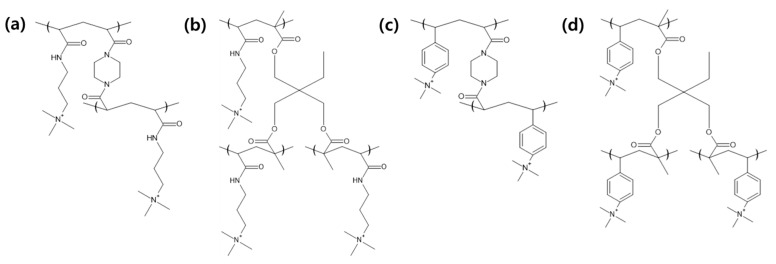
Chemical structure of anion-conducting polymers for (**a**) AE_2C, (**b**) AE_3C, (**c**) BE_2C, and (**d**) BE_3C.

Figure 6 shows the structure of ACPs polymerized with four combinations of electrolyte monomers and crosslinkers. Based on the predicted structural formula, the molecular weight per repeating unit is obtained, and then the theoretical IEC is calculated by dividing the number of moles of ion-exchange groups in the repeating unit by the molecular weight. The calculated theoretical IEC values are shown in Table 4. By calculating the theoretical IEC, the effective monomer mixing ratio can be predicted, but when compared to the measured IEC, the theoretical IEC may be different from the actually measured IEC because the value varies depending on the porosity or weight of the support actually used, and the polymerization yield is less than 100%. In addition, there is uncertainty due to experimental errors in acid–base analysis [38]. According to the molecular weight of the electrolyte monomer and crosslinker in Table 1, it should be noted that the IEC of the PFAEM is determined by the ratio of the decrease in the IEC value due to the increase in the weight of the membrane and the increase in the mole number of ion-exchange groups because the molecular weight of 3C is lower than that of 2C. This is because the crosslinker 3C has three crosslinking sites, so even though it has a higher molecular weight than the crosslinker 2C, it can exhibit a higher IEC because its contribution to the increase in the mole number of ion-exchange groups is greater. In this study, the electrolyte monomers and crosslinkers were chosen to ensure that the theoretical IEC values of the four PFAEMs were as similar as possible. The polymer chemical structures were designed to keep the deviation of the theoretical IEC values as low as possible, which were within 8%. Consequently, the measured IEC differences for the groups using the same crosslinker fell within the theoretical IEC deviation as expected. However, when comparing the measured IEC values of PFAEMs incorporating AE, a hydrophilic electrolyte monomer, with those incorporating BE, a hydrophobic electrolyte monomer, the IEC of the AE-based PFAEMs was approximately 20% higher than that of the BE-based PFAEMs. This discrepancy was significantly greater than the 1.6% difference predicted by the theoretical IEC. The underlying reason is that theoretical IEC values are calculated based on molar ratios and theoretical chemical structures. In practice, however, various physical factors such as steric hindrance, ion cluster size, and/or the distance between ion clusters in a hydrated state can influence ion movement within the ion exchange membrane [39].

**Figure 7:**
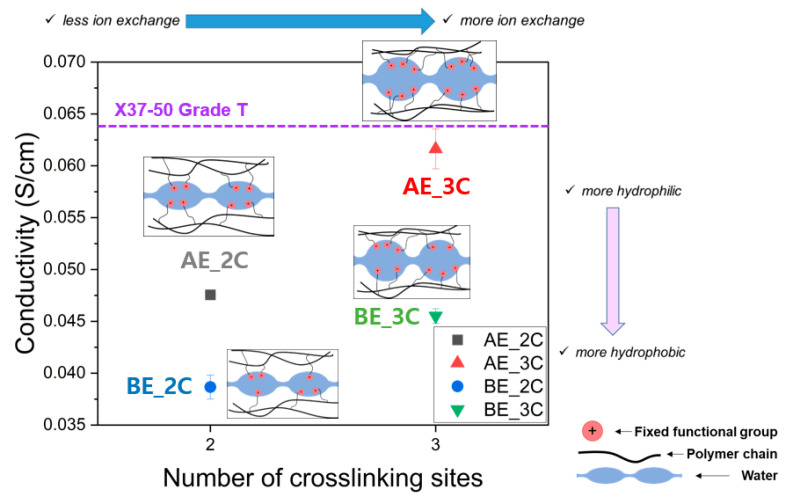
Ionic conductivity of the PFAEMs and the commercial AEM (the purple dotted line) and illustration of microstructure of the PFAEMs.

Similar to the IEC results, the ion conductivity measurements as shown in Figure 7 reveal that PFAEMs incorporating AE, a hydrophilic electrolyte monomer, exhibit a higher ion conductivity than those incorporating BE, a hydrophobic electrolyte monomer. Specifically, AE-based PFAEMs demonstrate 25% higher ion conductivity when using a 2C crosslinker and 41% higher ion conductivity when using a 3C crosslinker. This can be attributed to the higher measured IEC values of AE compared to BE, which directly influence the ion conductivity. In other words, the inclusion of hydrophilic AE in the polymer backbone likely facilitates the formation of ion clusters favorable for ion conduction. Moreover, while the measured IEC values show minimal variation with different crosslinkers within the same electrolyte monomer, ion conductivity increases by 30% when using AE and by 18% when using BE with a 3C crosslinker. This is believed to result from the 3C crosslinker, which features a high distribution of ion-exchange functional groups, promoting the formation of a phase-separated polymer structure in a hydrated state that enhances ion conduction as illustrated in Figure 7.

**Figure 8:**
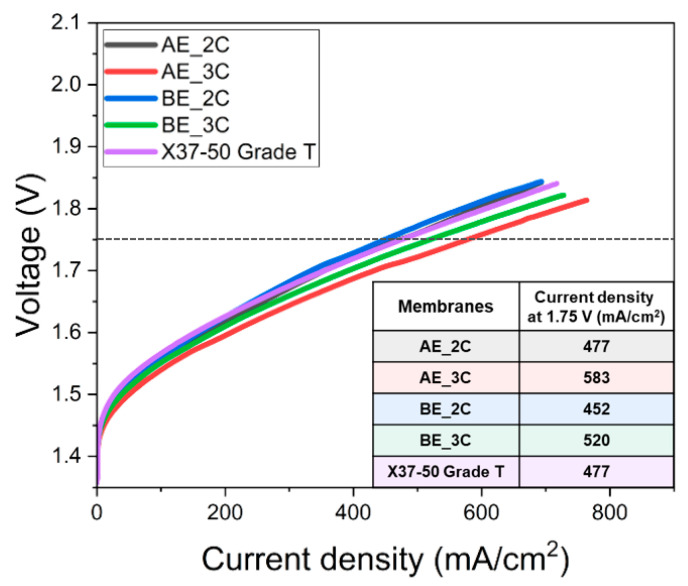
Performance curves of AEMWE using the PFAEMs and the commercial AEM (the inset table summarizes the current density values at 1.75 V for the PFAEMs).

The AEMWE performance results based on the properties of ACP are shown in Figure 8. At 1.75 V, the current density followed the order AE_3C > BE_3C > AE_2C > BE_2C for the PFAEMs. This aligns with the IEC and ionic conductivity data in Table 4 and Figure 7, where the trend AE_3C > AE_2C > BE_3C > BE_2C demonstrates high values, highlighting the significant influence of the hydrophilic properties of the polymer backbone. However, the number of crosslinking sites, which corresponds to the quantity of ion-exchange groups, have a greater effect on AEMWE performance. This relationship mirrors the trend observed in the contact angle measurements, where surface hydrophilicity follows the same order of AE_3C, BE_3C, AE_2C, and BE_2C. These results confirm that increased hydrophilicity of the PFAEM surface correlates with enhanced AEMWE performance. In the operational context of AEMWE, a 1 M KOH solution is supplied to the oxygen evolution electrode, where the solution interacts with the membrane surface. For improved AEMWE performance, low mass transfer resistance is essential at the membrane–solution interface, as hydroxyl ions generated at the hydrogen evolution electrode must be efficiently conducted through the AEM to the KOH solution. Thus, the hydrophilicity of the PFAEM surface is a critical factor influencing AEMWE performance.

**Figure 9:**
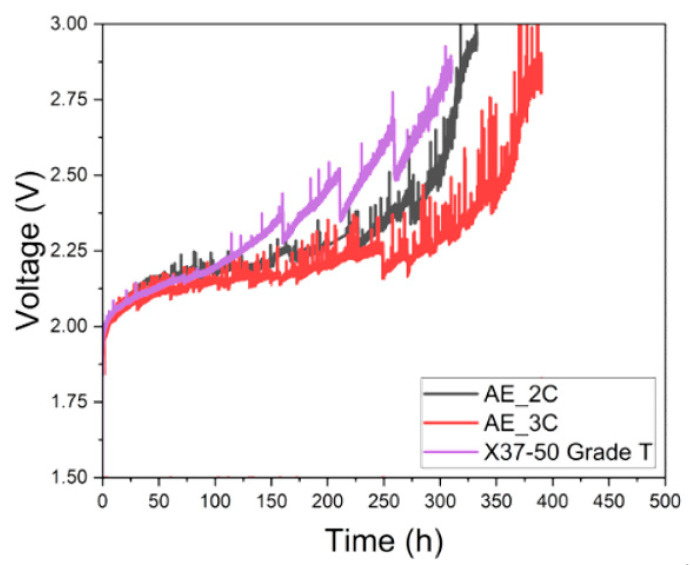
Variation in voltage at 0.5 A/cm^2^ as a function of AEMWE operation time for AE_2C, AE_3C, and the commercial AEM.

The durability of AEMWE was evaluated for four types of PFAEMs. However, due to the highly hydrophobic nature of the BE_2C membrane surface, voltage measurements were unstable, and the BE series PFAEMs were excluded from further evaluation. The durability results for the AE series PFAEMs (AE_2C and AE_3C) and a commercial AEM are presented in Figure 9. For AEMs containing quaternary ammonium groups as ion-exchange functional groups, degradation typically occurs through nucleophilic substitution or Hofmann elimination mechanisms. The observed voltage increase in Figure 9 is believed to result from these processes. Since the deactivation of ion-exchange groups drives the voltage increase during degradation, membranes with a higher number of ion-exchange groups per repeating unit are expected to demonstrate greater durability. This is supported by the measured IEC values, which follow the order AE_3C > AE_2C > X37-50 Grade T, mirroring the sequence of delayed voltage increases. These findings confirm that AEMs with a greater number of ion-exchange functional groups per repeating unit exhibit superior durability in AEMWE applications.

## 4. Conclusions

This study investigated the development and performance evaluation of four PFAEMs designed for AEMWE. The membranes, fabricated using AE and BE electrolyte monomers with 2C and 3C crosslinkers, exhibited distinct mechanical and electrochemical properties influenced by their hydrophilicity and IEC. AE-based PFAEMs consistently outperformed BE-based counterparts, demonstrating higher ion conductivity (up to 41% improvement) and superior AEMWE performance, with AE_3C achieving the highest current density at 1.75 V. These findings underscore the importance of hydrophilic components in enhancing ion transport and reducing interfacial mass transfer resistance. Durability testing confirmed that AE_3C exhibited superior stability, outperforming a commercial AEM due to its higher IEC and increased ion-exchange functional groups. This study also highlighted that mechanical strength, exceeding 14 times that of the commercial membrane, was achieved without compromising thickness or flexibility, a critical advancement for practical application. In conclusion, this study demonstrates that tailoring the chemical composition and crosslinking structure of ACPs within pore-filling membranes significantly enhances their performance and durability. Coupled with the advantage of seamless integration into roll-to-roll processes via rapid polymer curing reactions, these advancements pave the way for cost-effective and high-efficiency hydrogen production technologies.

## Figures and Tables

**Table 1:** Chemical structure, molecular weight, and chemical name of electrolyte, crosslinker, and photo-initiator.

Type	Chemical Structure	Molecular Weight (g/mol)	Chemical Name (Code)
Electrolyte	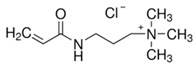	206.71	(3-acrylamidopropyl) trimethylammonium chloride (AE)
	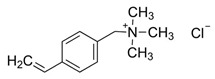	211.73	(Vinylbenzyl)trimethylammonium chloride (BE)
Crosslinker	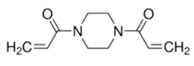	194.23	1,4-bis(acryloyl) piperazine (2C)
	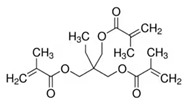	338.40	Trimethylolpropane trimethacrylate (3C)
Photo-initiator	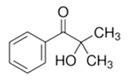	164.20	2-hydroxy-2-methylpropiophenone (-)

**Table 2:** Tensile strength and elongation at break for PE substrate, PFAEMs, and commercial AEM.

Sample		Tensile Strength (MPa)	Elongation at Break (%)
PE substrate		106.4	119.7
PFAEM	AE_2C	121.5	88.32
	AE_3C	118.8	94.99
	BE_2C	113.3	50.26
	BE_3C	137.0	86.05
X37-50 Grade T		8.30	2.52

**Table 3:** Ion transport number of PFAEMs and commercial AEM.

Sample		Transport Number
PFAEM	AE_2C	0.991
	AE_3C	0.996
	BE_2C	0.996
	BE_3C	0.999
X37-50 Grade T		0.972

**Table 4:** Theoretical and experimental IEC of the PFAEMs and the commercial AEM.

Sample		Theoretical IEC (meq/g)	Experimental IEC (meq/g)
PFAEM	AE_2C	2.50	1.74
	AE_3C	2.66	1.80
	BE_2C	2.46	1.43
	BE_3C	2.62	1.53
X37-50 Grade T		-	1.40

## Data Availability

The raw data supporting the conclusions of this article will be made available by the authors on request.
